# Optogenetic inhibition of ventral hippocampal neurons alleviates associative motor learning dysfunction in a rodent model of schizophrenia

**DOI:** 10.1371/journal.pone.0227200

**Published:** 2019-12-31

**Authors:** Zheng-li Fan, Bing Wu, Guang-yan Wu, Juan Yao, Xuan Li, Ke-hui Hu, Zhen-hua Zhou, Jian-feng Sui

**Affiliations:** 1 Department of Physiology, College of Basic Medical Science, Army Medical University, Chongqing, P.R. China; 2 Experimental Center of Basic Medicine, College of Basic Medical Science, Army Medical University, Chongqing, P.R. China; 3 Department of Rehabilitation, Suining Central Hospital, Suining, P.R. China; 4 Department of Neurology, First Affiliated Hospital of Army Medical University, Chongqing, P.R. China; Technion Israel Institute of Technology, ISRAEL

## Abstract

Schizophrenia (SZ) is a serious and incurable mental disorder characterized by clinical manifestations of positive and negative symptoms and cognitive dysfunction. High-frequency deep brain stimulation (DBS) of the ventral hippocampus (VHP) has been recently applied as a therapeutic approach for SZ in both experimental and clinical studies. However, little is known about the precise mechanism of VHP-DBS treatment for SZ and the role of hippocampal cell activation in the pathogenesis of SZ. With optogenetic technology in this study, we tried to inhibit neuronal activity in the VHP which has dense projections to the prefrontal cortex, before measuring long stumulus-induced delay eyeblink conditioning (long-dEBC) in a rodent model of SZ. Rats were administrated with phencyclidine (PCP, 3 mg/kg, 1/d, ip) for successive 7 days before optogenetic intervention. The current data show that PCP administration causes significant impairment in the acquisition and timing of long-dEBC; the inhibition of bilateral VHP neurons alleviates the decreased acquisition and impaired timing of longd-dEBC in PCP-administered rats. The results provide direct evidence at the cellular level that the inhibition of VHP neuronal cells may be a prominent effect of hippocampal DBS intervention, and increased activity in the hippocampal network play a pivotal role in SZ.

## Introduction

Schizophrenia (SZ) is a severe, chronic, refractory mental disorder characterized by functional derangements in multiple brain regions, such as hippocampus and prefrontal cortex (PFC)[1]. High-frequency deep brain stimulation (DBS) is an effective alternative therapy to treat patients with motor and mental dysfunctions such as Parkinson’s disease, depression, and obsessive-compulsive disorder[2–6]. Encouragingly, DBS has recently been attempted as a therapeutic approach for SZ[2, 7–10]. DBS of the ventral hippocampus restores deficits in auditory evoked potentials in the infralimbic cortex and the mediodorsal thalamic nucleus in a rodent developmental disruption model of SZ[2]. DBS of the medial septum and the nucleus accumbens alleviated prepulse inhibition (PPI) deficits and abnormal psychosis-relevant behaviour in ketamine-treated rats[10]. DBS delivered to the mPFC or nucleus accumbens of the adult progeny of poly I:C-treated dams improves deficits in sensorimotor gating (measured by PPI) and attention selectivity (reflected by latent inhibition)[9]. A clinical trial for DBS in patients with treatment-resistant SZ demonstrated clinical improvement in both positive and negative symptoms after 4 weeks of nucleus accumbens DBS[8]. However, although the effect of high frequency DBS was assumed to inhibit local neuronal activities in SZ treatment, which is comparable to the local lesion effects of ablative surgery[11], direct evidence of the precise celellar target of DBS treatment for SZ has been scant, which may be of significance for the further application of DBS in SZ therapy.

The substantial challenge of elucidating the mechanism of DBS has been attributed to the high heterogeneity in the stimulated brain tissues in which the electrodes are placed. During the course of DBS, neuronal cellular bodies, or the afferent fibers originating from remote brain areas, or even the local glial cells might be either activated or depressed by the electrical current[5, 6]. Classical experimental intervention approaches are unable to dissect these effects due to their inherent methodological limitations of low spatial resolution and low temporal resolution during intervention. However, with the development of optogenetics which has the distinct advantages of being able to isolate interventions to a single neural component, comprehensively elucidating the neural mechanisms underlying DBS treatment in a series number of neurological and psychiatric disorders is becoming feasible. Optogenetics allows targeted and fast intervention in precisely defined brain cells by delivering optical control at the millisecond timescale and with the cell type-specific precision needed[12]. By systematically driving or inhibiting distinct relevant elements in freely moving parkinsonian rats with this technique, the therapeutic mechanism of DBS delivered to the subthalamic nucleus has been clarified for treating Parkinson’s desease, i.e., the selective stimulation of the targeted afferent axons projecting to this region from the motor cortex[13], providing a successful example for exploring the mechanisms of the curative effect of DBS applied in neurological diseases. Excessive hippocampal output is thought to be responsible for the hyperactivation of the dopamine system resulting in positive symptoms and cortical disruption associated with cognitive deficits in SZ[14]. In the present study, selective optogenetic inhibition was delivered to neurons in the ventral hippocampus (VHP) in a rodent model of phencyclidine (PCP)-induced SZ, followed by the assessment of associative motor learning behaviour to explore the intervention target of hippocampal DBS responsible for SZ treatment.

PCP is a psychotomimetic drug and has been demonstrated to elicit a range of schizophrenic symptoms in healthy persons, including not only positive and negative symptoms but also cognitive disorders[15]. The administration of PCP in animals has been regarded as the most reliable pharmacological model of SZ[16]. Relevant studies reveal that long-lasting excitation of neurons in the prefrontal cortex (PFC) after systemic administration of PCP may be responsible for the development of schizophrenic behavioural disorders[17, 18], whereas ventral hippocampus, which has excitatory projections to the PFC[19], seems to play a pivotal role in triggering PCP-induced prefrontal neuronal activation; DBS delivered to the ventral hippocampus can restore deficits in auditory evoked potentials in a rodent developmental disruption model of SZ[2]. In addition, it has already been observed that patients with SZ showed functional and structural abnormalities in the hippocampus[20]. However, there is still a lack of direct evidence at the cellular level that increased activities in the hippocampal network play a pivotal role in SZ.

Eyeblink conditioning (EBC) is one of the most thoroughly elucidated models of associative motor learning[21, 22]. EBC training involves paired presentations of a conditioned stimulus (CS, usually a tone or a light) and an unconditioned stimulus (US, usually a periorbital shock or an air puff). If the CS starts before the US and the two stimuli co-terminate, the delay EBC (dEBC) paradigm will be established; when the CS and US are separated in time by a trace interval, the behaviour of trace EBC (tEBC) will be learned. It has been well established that simple dEBC (dEBC with optimal parameters) is mediated necessarily and sufficiently by the cerebellum-brainstem circuit[23], whereas EBC with high task difficulty, including tEBC or dEBC with non-optimal parameters, such as having a much longer or much weaker CS, involves multiple cortical and subcortical structures in addition to the cerebellum-brainstem circuit, such as the hippocampus[19, 24–25]. In recent years, simple dEBC deficits have been observed in SZ in some studies, and efforts have been made to consider these deficits as a possible biomarker for neurodevelopmental disorders, but conflicting results exist[26]. Given that long-dEBC is hippocampal-dependent and schizophrenic patients exhibit evidence of hippocampal dysfunction, we hypothesized that it may be possible to modulate or normalize long-dEBC by optogenetically tuning the hippocampus in an SZ rodent model. In this experiment, rats were administrated with 3 mg/kg of PCP for 7 days (1/d, ip), and then long-dEBC behaviour with a 3.3 second CS duration was measured after the successive optogenetic inhibition of ventral hippocampal neuronal activities for 1 week. Cognitive deficits are considered the primary cause of most behavioural disabilities associated with SZ [27–29]. Our data showed that PCP administration impaired long-dEBC behaviour and that the inhibition of VHP neurons ameliorated long-dEBC deficits in SZ-like rats. This result suggests that hippocampal DBS intervention in SZ involved the inhibition of hippocampal network and that activated hippocampal cells were involved in the pathogenesis of SZ.

## Materials and methods

### Animals

Fifty-eight healthy male Sprague Dawley (SD) rats weighing between 300g and 350 g were individually housed in standard stainless steel cages (50×30×35cm) with free access to water and food, and maintained on a 12-h light/dark cycle. Seven rats that lost a headstage or had inaccurate virus injection or fiber-optic cannula placements had been excluded from the study. Treatments, behavioral experiments were performed during the light period. All procedures were approved by the Animal Care Committee of the Army Medical University and were performed in accordance with the principles outlined in the NIH Guidelines for the Nursing and Use of Laboratory Animals.

According to the type of infected virus and treated drugs, all rats were randomly divided into four groups, namely (1) saline-treated group (n = 13): the rats were intraperitoneally injected with physiological saline for 7 successive days; (2) PCP-treated group (n = 10): the rats were intraperitoneally injected with PCP for 7 successive days; (3) PCP-eNpHR group (n = 12): the rats were micro-injected with pAAV2/9-hSyn-eNpHR3.0-EYFP bilaterally in VHP, and 2 weeks later, intraperitoneally injected with PCP for 7 days; (4) PCP-EYFP group (n = 16): rats were micro-injected with pAAV2/9-hSyn-EYFP in VHP, and 2 weeks later, intraperitoneally injected with PCP for 7 days.

### Animal anesthesia

All SD rats were anesthetized by intraperitoneal injection of 3% sodium pentobarbital (35 mg/kg, i.p., SERVA, Denmark) and then supplemented with intramuscular injection of atropine sulfate (1.5 mg/kg, i.p., TaiJi, Southwest Pharmaceutical Co., Ltd., China) to prevent asphyxia caused by increased airway secretions. The depth of anesthesia was tested by measurement of cornea reflex and body pain reflex. Supplementary dosage of anesthetic would be added when necessary. All possible efforts such as adequate anesthesia, soft manipulations had been made to alleviate animal suffering. Animals were allowed to recover 5–7 days after a surgery.

### Virus injection

**Fig 1:**
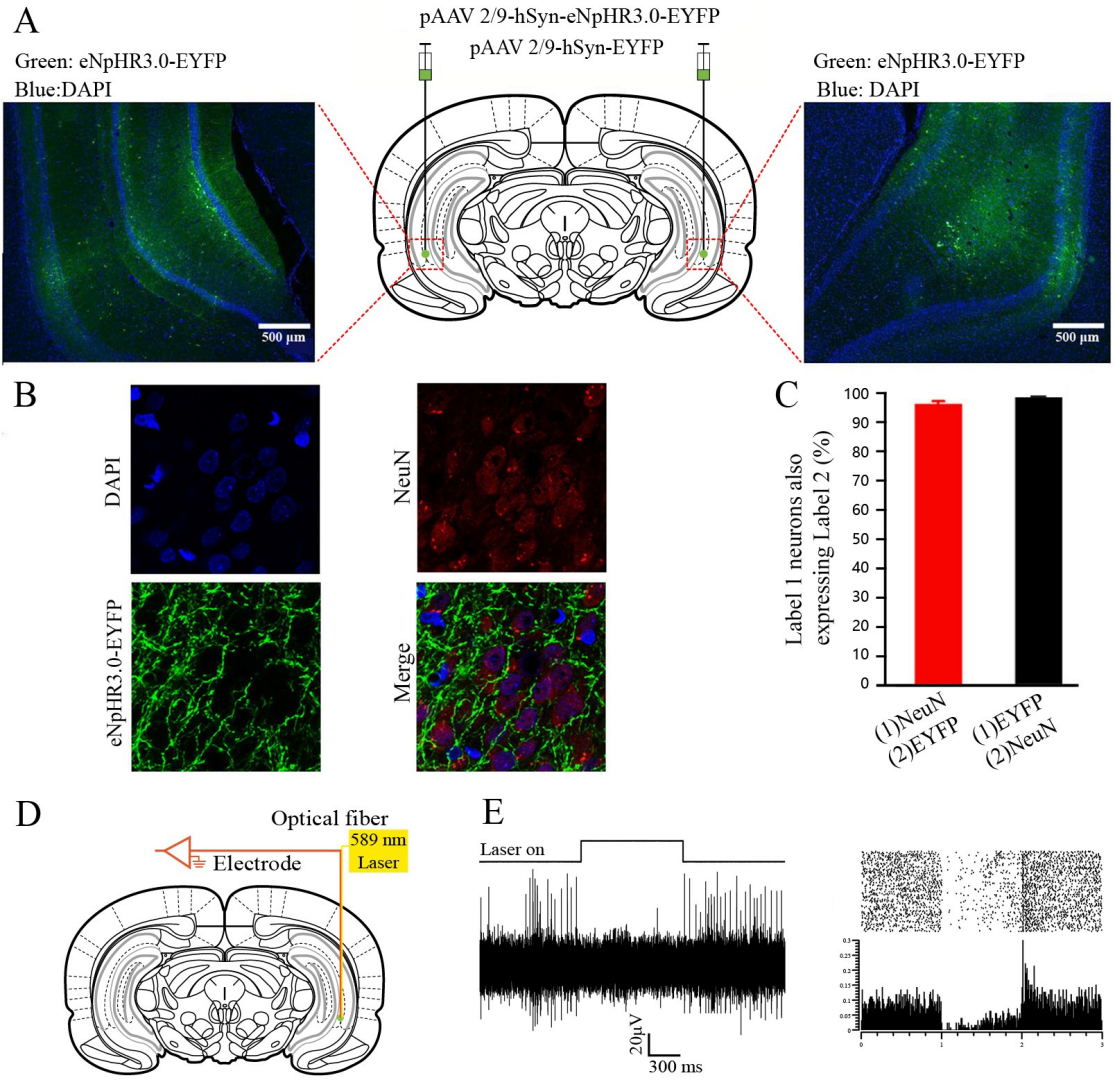
Labelling and optogenetic inhibition of the bilateral VHP neurons. (A) Schematic diagram of injection of pAAV 2/9-hSyn-eNpHR3.0-EYFP or pAAV2/9-hSyn-EYFP targeting the bilateral VHP, and the example of eNpHR3.0-EYFP expression in the bilateral VHP. (B) Representative images showing cell-specific eNpHR3.0-EYFP expression (green) in neurons (red) of VHP. Scale bar, 20μm. (C) Statistics of expression in neurons (843 cells, from 7 rats). (D) Schematic diagram of optogenetics technology inhibits cell activity in vivo and extra-cellular recordings. (E) 589-nm yellow light can inhibit the spontaneous firing of neuron expressing eNpHR3.0-EYFP.

After anesthesia SD rats were fixed in a brain stereotaxic apparatus (Model 942, David Kopf Instruments, Tujunga, California, USA) for surgery. The virus was injected using a glass micropipette (tip diameter 10–20 μm) attached to a 5 μL Hamilton microsyringe (51189, Stoelting, Wood Dale, Illinois, USA). The injection rate (0.05 μL/min) was controlled by a stereotaxic microsyringe pump (53311, Stoelting Company, USA). A small craniotomy was performed to prepare small holes on the surface reflection of the bilateral hippocampus and the virus was delivered into the bilateral VHP. Only one location for each hippocampus was injected. Rats were micro-injected with 0.3 μL of pAAV2/9-hSyn-eNpHR3.0-EYFP [Virus titers: 7.18 × 10^12^ genome copy (GC)/mL] or the control virus pAAV 2/9-hSyn-EYFP [Virus titers: 1.17×10^13^ GC/mL] into both left and right VHP [anteroposterior (AP) - 5.8 mm from bregma, mediolateral (ML) ± 5.5 mm, dorsoventral (DV) - 7.0 mm] (Fig 1A). After injection, the needle was left in place for 5 additional minutes and then slowly withdrawn, and after sufficient hemostasis, the skin was sutured.

### Immunohistochemistry

For immunohistochemistry experiments, after the experiment was completed, the virus-infected rats were deeply anesthetized with an overdose of sodium pentobarbital (120 mg/ kg) intraperitoneally and perfused transcardially with 500 ml physiological saline followed bycold 4% polyformaldehyde solution (PFA; prepared in 0.1 M of phosphate buffer, pH 7.4). The brains were removed from the skull and stored in 4% PFA at 4°C for 24 h, and then transferred to a 30% sucrose solution at 4°C for 48 h. Then the brain tissues were encapsulated with O.C.T cryosurgery agent (Sakura Finetek Company, USA) and were cut into 30 μm-thick coronal sections on a freezing microtome (CM3050S, Leica, Germany) and collected in cold phosphate buffer saline (PBS, 0.01 M, pH 7.4). For immunostaining, each slice was placed in PBST (PBS + 0.3% Triton X-100) with 2% normal bovine serum for 1 h incubated with primary antibody at 4°C for 24 h (Mouse anti-NeuN 1:500, PC213L, Merck Millipore, Billerica, MA, USA). Slices then underwent three wash steps for 10 min each in PBS-T, followed by 1 h incubation with secondary antibody (Goat anti-mouse conjugated to Alexa Fluou568, 1:500, Invitrogen). Slices were then incubated for 5 min with DAPI (1:2000, Sigma-Aldrich, St. Louis, MO, USA) and underwent three more wash steps of 10 min each in PBS-T, followed by mounting and cover slipping on microscope slides. Confocal fluorescence images were acquired on a Carl Zeiss LSM 780 scanning laser microscope (Germany) using a 40 × oil immersion objective.

### Electrophysiological verification of optogenetics

Three-four weeks after virus injection, the virus-infected rats used for electrophysiological verification were anesthetized and their heads were fixed on the stereotaxic apparatus (Model 942, David Kopf Instruments, Tujunga, California, USA) after routine skin preparation. An optrode consisting of a fiber-opticconnula (200 μm core diameter, 0.39 numerical aperature, FT200EMT, Torlabs, Newton, New Jersey, USA) with a multi-wire electrode tightly coupled with an optical fiber, with the tips of the electrodes extending approximately 400 μm beyond the tip of optical fiber was used for simultaneous optical stimulation and extracellular recordings. Electrodes were made of 16 individually insulated nichrome wires (17.78 μm inner diameter, 761000, A-M Systems, Sequim, WA, USA), attached to a 20 pin connector. The optrode was pushed slowly to the right VHP (- 5.8 mm AP, 5.5 mm ML, - 7.0 mm DV) by a hydraulic micromanipulator (IVM-1000, Scientifica, UK). The optic fiber was connected to the 589 nm laser (YL589T6-100FC, Laser & Optics, Shanghai, China) controlled by a pulse stimulator (Master-9, A.M.P.I., Jerusalem, Israel) (Fig 1D). After light-responsive cells were detected, a series of light stimuli were conducted: 100 epochs of 1000-ms light pulse trains (589 nm, 3 mW), separated by a random interval of 20–40s and an average time interval of 30 s. The electrophysiological signals were band-pass filtered (0.3–5 kHz) using an 16-channel microelectrode amplifier (model 3600, A-M Systems, Sequim, WA, USA) and recorded by a data acquisition system (Powerlab 16/35, ADInstuments, New South Wales, Australia) with a sampling rate of 20 kHz. NeuroExplorer 4 (MicroBrightField, Williston, VT, USA) software was used to analyze the neurophysiological data (Fig 1E).

### Implantation of optical fibers and electrodes for recording and stimulation

**Fig 2:**
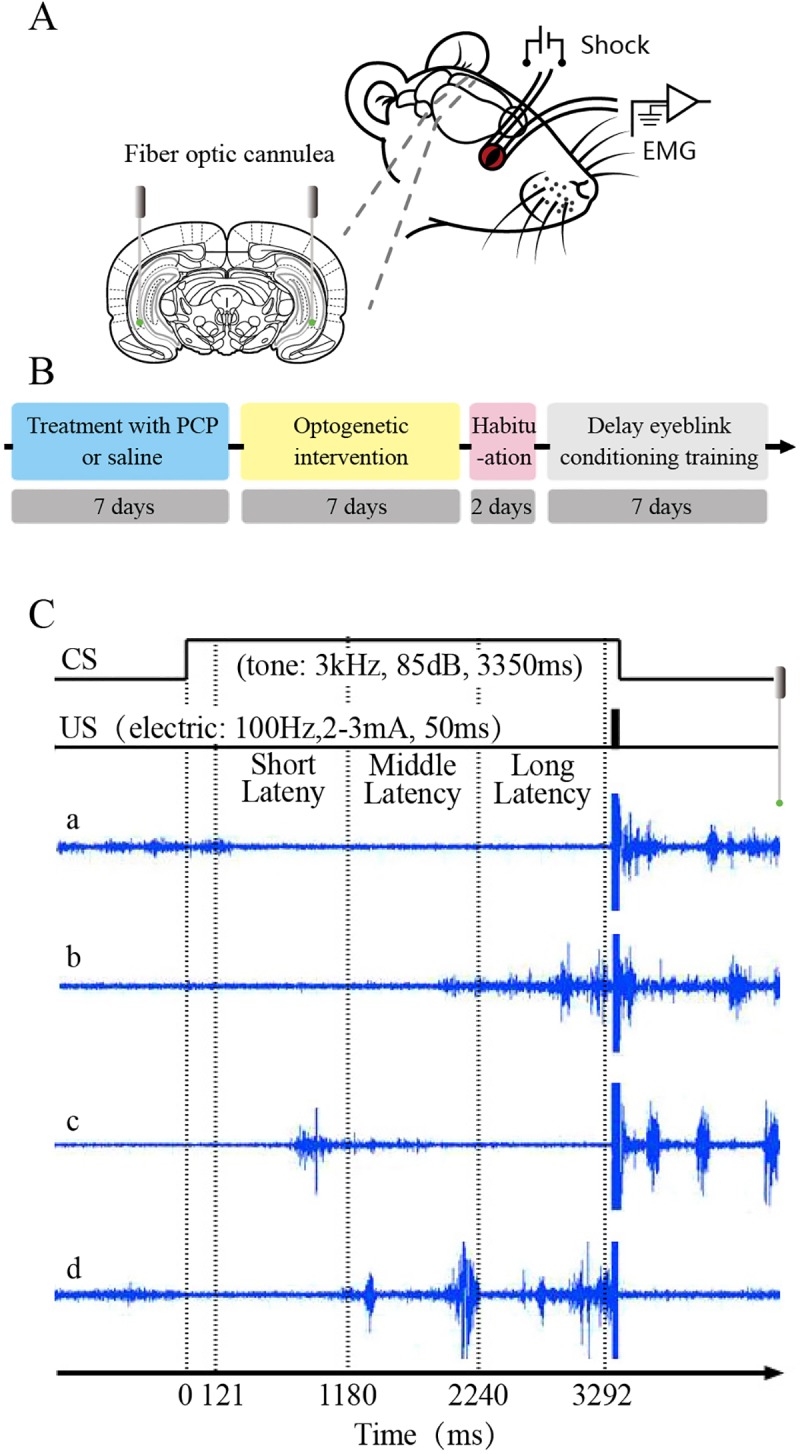
The behavioral training paradigm. (A) For behavioral training, four electrodes were implanted in the upper left eyelid, two for electrical stimulation as the US and the other two for recording the EMG activity of the orbicularis oculi (O.O.) muscle. The optical fiber buried in bilateral VHP. (B) Treatment and training procedure of each rat. (C) Experimental design and temporal relationship between the conditioned stimulus (CS), unconditioned stimulus (US), and analysis periods for the eye-blink conditioning (EBC). The CS was presented 3300 ms before the US onset and remained on while the 50-ms shock was delivered and they terminated together. In each trial, eye blinks with short-latency (121–1180 ms), with middle-latency (1181–2240 ms) and long-latency (2241–3292 ms) were analyzed respectively.

All of the rats used for behavioral training were anesthetized and fixed in a brain stereotaxic apparatus (Model 942, David Kopf Instruments, Tujunga, California, USA) for surgery at 3–4 weeks after virus injection. After complete anesthesia in rats, optical fibers (200 μm core diameter, 0.39 numerical aperture, FT200EMT, Torlabs, Newton, New Jersey, USA) were pushed slowly to the bilateral VHP (- 5.8 mm AP, 5.5 mm ML, - 7.0 mm DV) through the small cranial holes previously made for virus injection, and four electrodes made of insulated stainless steel wires (76.2 μm inner diameter, 790900, A-M Systems, Sequim, WA, USA) were implanted in the upper eyelid of the left eye (Fig 2A). One pair of electrodes was implanted into the ipsilateral orbicularis oculi (O.O.) muscle to record the electromyography (EMG) activity, and the other pair was implanted subdermally caudal to the left eye to deliver the electrical stimulation (electric shock: 100 Hz, 1–2 mA, 50 ms). The electrode tips were bent as a hook to facilitate a stable insertion in the upper eyelid. Moreover, a bare silver wire (0.1 mm in diameter) was connected to four stainless steel skull screws as a ground. The seven wires were connected to an 8-pinmini-strip connector. The mini-strip connector and optical fiber cannula were cemented to the skull with dental cement. After the surgery, the animals were allowed 1 week of recovery.

### Drugs administration, optogenetic intervention, and behavioral procedures

Prior to behavioral acquisition training rats were treated with daily intraperitoneal injection of PCP (Chemsky [Shanghai] international Co., LTD, Shanghai, China) at dosage of 3 mg/kg, or equal volume of physiological saline (Taiji Southwest Pharmaceutical C., Ltd., Chongqing, China), once a day for 7 consecutive days. After the injection of PCP, the virus-infected rats received continuous 589-nm light (3 mW, 5 min) on both sides of VHP 3 times a day for 7 consecutive days. The duration of each light on was 5 min, and the interval between two lights also was 5 min.

Prior to behavioral acquisition training, all rats firstly underwent a two-day habituation periods in a plastic box (35× 25 × 20 cm) (Fig 2B). No stimulation was given to the rats, and they were adapted to about 60 min each day. Long-delay eyeblink conditioning (long-dEBC) was carried out to be the behavioral paradigm (Fig 2C). During behavioral training, the conditioned stimulus (CS) was a 3-kHz, 85-dB, and 3350-ms pure tone. All sound stimuli were delivered by two electrostatic speakers fixed on the inner wall of the training box. A sound-level meter (type2240, Brüel & Kjær) was used to calibrate the intensity of the CS tone. The unconditioned stimulus (US) was a 100-Hz, 1–2 mA, and 50-ms periorbital electrical shock (1-ms pulse duration, square, cathodal pulse), delivered by a stimulus isolator (ISO-Flex, A.M.P.I., Jerusalem, Israel), controlled by a pulse stimulator (Master-9, A.M.P.I., Jerusalem, Israel). The intensity of the shock US was carefully calibrated to give the minimal current required to elicit a discrete eyeblink response. The CS and US intensity were set before the first acquisition session and were not changed during the rest of the experiment. For the delay conditioning paradigm, the US terminated simultaneously with the offset of the CS (3350 ms duration), and the inter-stimulus interval was 3300 ms. In the daily acquisition session, we administered 12 blocks of 120 long-dEBC trials at an inter-trial interval of 25–40 s. Each block consisted of 10 trials, each of which comprised 9 CS-US paired trials and 1 CS-alone trial.

### Behavioral data analysis

EMG activity of the O.O. muscle and the LFP signals were bandpass filtered (0.1–1 kHz and 0.3–100 Hz, respectively) using a16-channel differential amplifier (model 3500, A-M Systems, Sequim, WA, USA) and acquired with a data acquisition system (Powerlab 16/35, ADInstuments, New South Wales, Australia) with a sampling rate of 10 kHz.

EMG data were analyzed off-line for quantification of conditioned responses (CRs) with the help of a custom program based on Matlab. The collected EMG data were full-wave rectified and integrated with a 1-ms time constant. The integrated EMG activity was calculated to the standard score compared to the mean of the baseline activity for the 0–300 ms before the CS onset in each trial. The mean plus 4 times standard deviation (SD) of the standard EMG activity during the baseline period of each trial was defined as the trial threshold. If the standard EMG amplitude during baseline period exceeded the trial threshold and lasted>10 ms, the trial was regarded as a hyperactivity trial and excluded from further analysis. Moreover, a trial was considered to contain the CR if the standard EMG amplitudes exceeded the trial threshold and lasted>10 ms during the period of 121–3292 ms after the CS onset. Each trial data was divided into three time windows for statistical analysis (Fig 2C). They were short-latency window (121–1180 ms), middle-latency window (1181–2240 ms) and late-latency window (2241–3292 ms), respectively. The percentage of CR (CR%) was defined as the ratio of the number of trials containing the CR to the total number of valid trials. The CR peak amplitude was defined as the maximum amplitude change from the baseline during the CR period. The CR start latency was defined as the time interval from the CS onset to the time when the standard EMG amplitude first met the criterion for a CR. Moreover, the CR peak latency was defined as the time interval from the CS onset to the peak of the EMG amplitude. Note that only trials containing CRs were selected for analysis of CR peak amplitude, CR onset latency, and CR peak latency. In addition, data from animals were excluded from the analysis under the following circumstances: (1) the headstages dropped out of the skull before the end of the experiments and lead to incomplete recording of the experimental data; (2) the sites of virus injection, or the placements of fiber-optic cannula were not in the target areas.

### Histology

After behavioral experiments, rats were deeply anesthetized with an overdose of sodium pentobarbital (120 mg/ kg) intraperitoneally and perfused transcardially with 500 ml physiological saline followed by cold 4% PFA. The brains were removed from the skull and stored in 4% PFA at 4°C for 24 h, and then transferred to a 30% sucrose solution at 4°C for 48 h. Then the brain tissues were cut into 30 μm-thick coronal sections on a freezing microtome (CM3050S, Leica, Germany) and collected in cold phosphate buffer saline (PBS, 0.01 M, pH 7.4). Slices underwent 3 more wash steps of 10 min each in PBST, followed by mounting and coverslipping on microscope slides. The extents of virus expression and placements of fiber-optic cannula, were carefully checked, and their images were acquired using an Olympus BX53F fluorescence microscope (Japan) using a 2 × air objective.

### Statistical analysis

All the data were expressed as the mean ± standard error of the mean (SEM). Statistical significance was determined by a least significant difference (LSD) post hoc tests, following a two-way repeated measures analyses of variance (ANOVA) and a separate one-way repeated measures ANOVA using the SPSS for Windows package(v. 18.0). A value of P < 0.05 was considered to be statistically significant.

## Results

### Impaired acquisition of long-dEBC in PCP-treated rats

**Fig 3:**
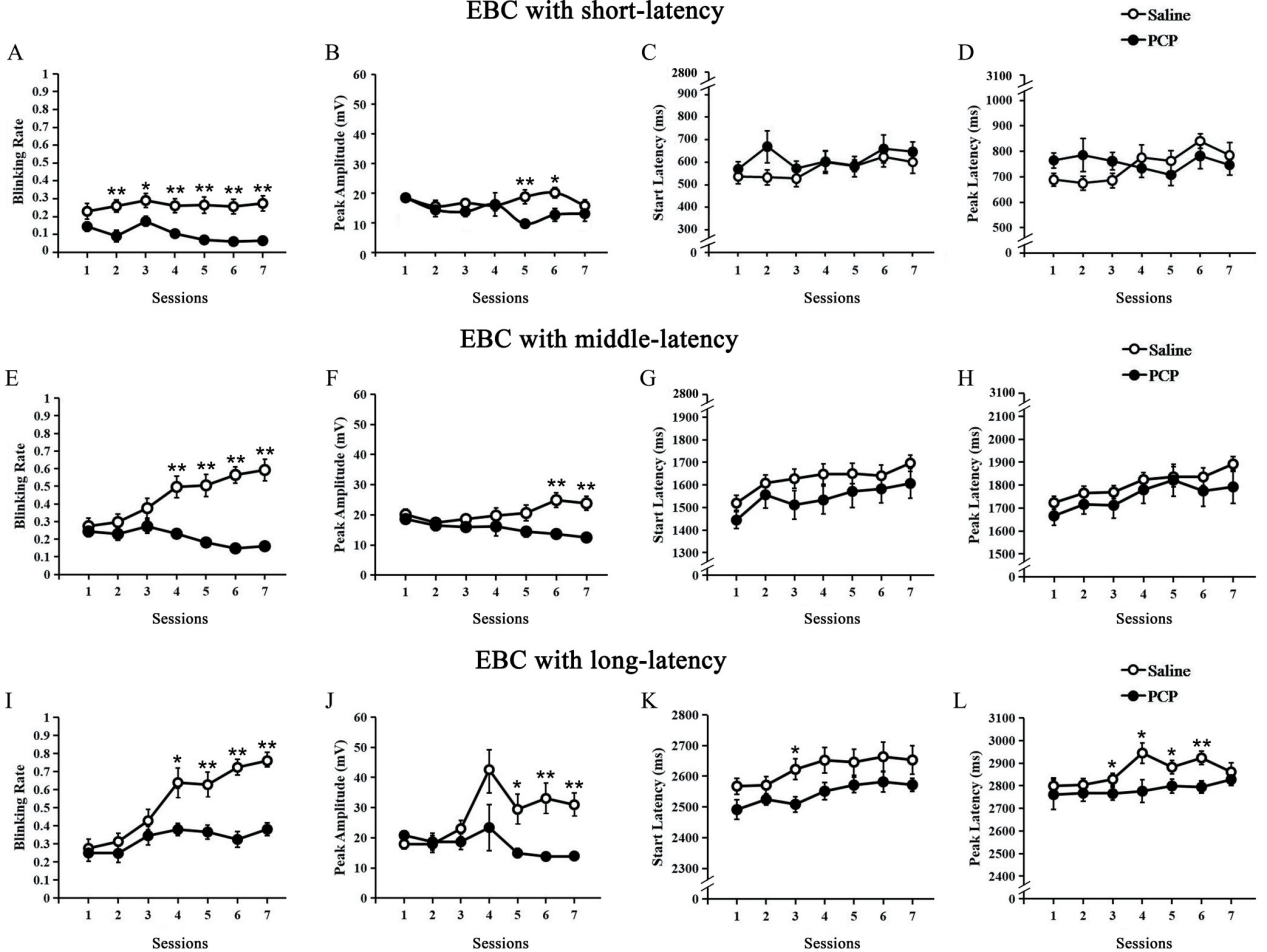
Effects of PCP on delay eyeblink conditioning (dEBC) in rats. (A–D) Results of dEBC with short-latency. (A) Percentage of CR (CR%) in paired trials in saline group (n = 13) and PCP group (n = 10) across the 7 sessions. (B) The CR peak amplitude, (C) CR start latency and (D) CR peak latency, of the saline and PCP group during the 7 sessions. (E–H) Results of dEBC with middle-latency. (E) Percentage of CR (CR%) in paired trials in saline and PCP group with middle-latency. (F) The CRpeak amplitude, (G) CR start latency and (H) CR peak latency, of the saline and PCP group with middle-latency during the 7 sessions. (I–L) Results of dEBC with long-latency. (I) Percentage of CR (CR%) in paired trials in saline and PCP group with long-latency. (J) The CR peak amplitude, (K) CR start latency and (L) CR peak latency, of the saline and PCP group with middle-latency during the 7 sessions. Data of bars are represented as mean ± standard error of the mean (SEM). Error bars indicate the SEM. *P<0.05, **P<0.01.

To address the possibility of establishing the long-dEBC with CS length of 3350 ms in rats, and whether the establishment of long-dEBC could be affected by PCP, we trained the rats with saline treated or PCP treated on dEBC paradigm (Fig 3). The rats of saline-treated group showed a significantly increases in blinking rate within middle- and long-latency windows across sessions 1–7 (Fig 3E and 3I). In contrast, there were no progressive increases in blinking rate of PCP-treated group, and it was significantly lower than the saline-treated group with short-, middle- and long-latency windows (Fig 3A, 3E and 3I). The peak amplitude reflecting the behavioral intensity and the peak latency representing the behavioral timing accuracy of CRs were all analyzed in the current study. The peak amplitude of saline-treated group was significantly higher than PCP-treated group in later sessions (5, 6, or 7) across 3 time windows (Fig 3B, 3F and 3J); the start latency in session 3 and the peak latency in sessions 3–6, of CR with long-latency, were significantly decreased in PCP-treated group than in saline-treated group, suggesting the impaired behavioral timing. These results suggested that long-dEBC behavior model could be successfully established in rats, and it would be impaired by PCP administration.

### Optogenetic inhibition of VHP neurons alleviated the long-dEBC deficits in PCP-treated rats

**Fig 4:**
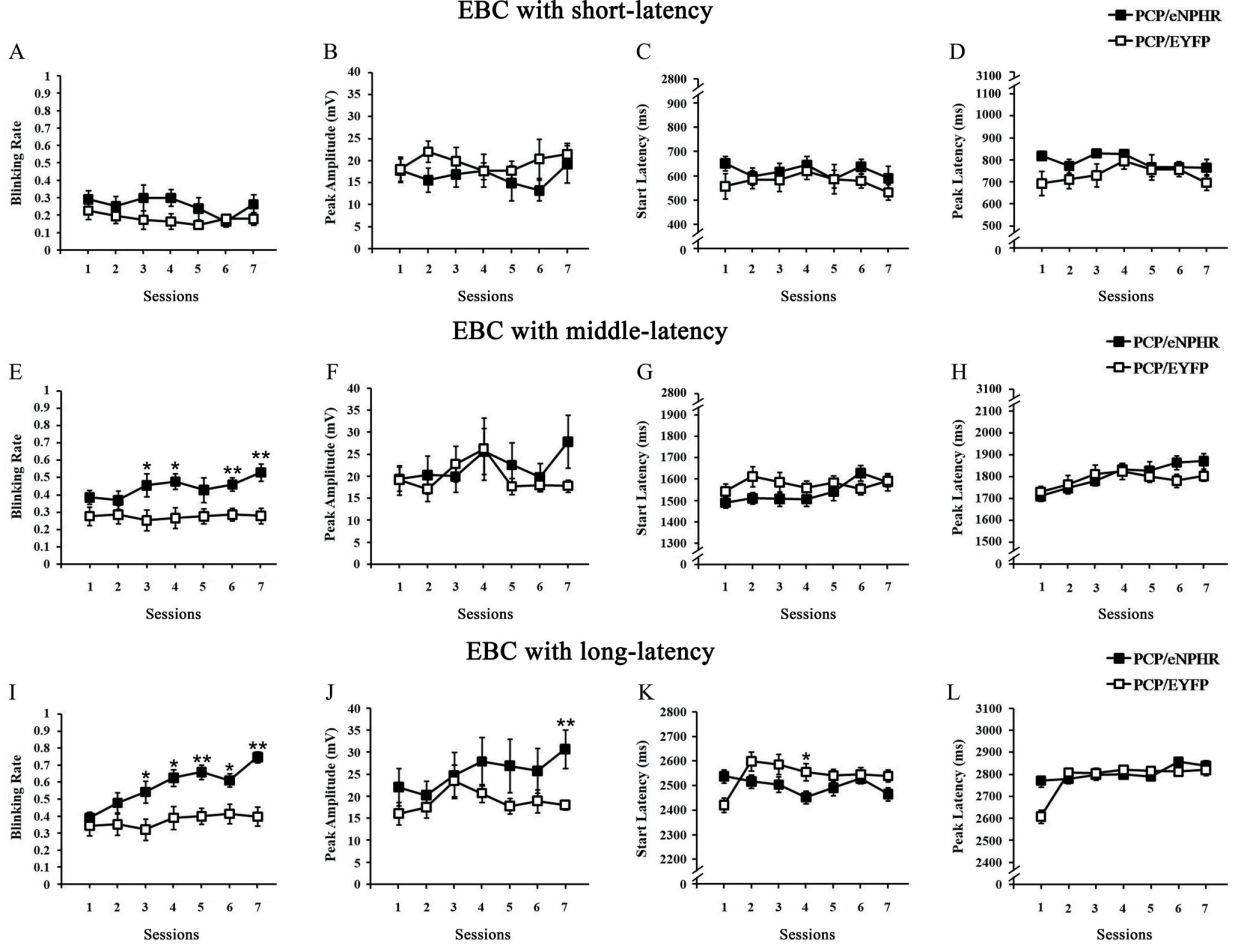
Effects of optogenetic inhibition of VHP neurons on dEBC in PCP-treated rats. (A–D) Results of dEBC with short-latency. (A) Percentage of CR (CR%) in paired trials in PCP-eNpHR group(n = 12) and PCP-EYFP group (n = 16) across the 7 sessions. (B) The CR peak amplitude, (C) CR start latency and (D) CR peak latency, of the PCP-eNpHR and PCP-EYFP group during the 7 sessions. (E–H) Results of dEBC with middle-latency. (E) Percentage of CR (CR%) in paired trials in PCP-eNpHR and PCP-EYFP group with middle-latency. (F) The CR peak amplitude, (G) CR start latency and (H) CR peak latency, of the PCP-eNpHR and PCP-EYFP group with middle-latency during the 7 sessions. (I–L) Results of dEBC with long-latency. (I) Percentage of CR (CR%) in paired trials in PCP-eNpHR and PCP-EYFP group with long-latency. (J) The CRpeak amplitude, (K) CR start latency and (L) CR peak latency, of the PCP-eNpHR and PCP-EYFP group with middle-latency during the 7 sessions.Data of bars are represented as mean ± standard error of the mean (SEM). Error bars indicate the SEM. *P<0.05, **P<0.01.

To access the requirement of the VHP neurons for dEBC in PCP-treated rats, we use optogenetic approach to suppress the activities of neurons in VHP for 7 consecutive days before behavioral training. Firstly plasmid adeno-associated virus (pAAV) or control viruses were injected into the bilateral hippocampus. The viruses encode either the third-generation chloride pump halorhodopsin which was fused in-frame to enhanced yellow fluorescent protein (eNpHR3.0-EYFP) or only EYFP[30]. Both target genes are under control of the human synapsin I (hSyn) promoter (Fig 1A). The efficacy and specificity of the virus transfection were validated in vitro (Fig 1B). About 96.15 ± 0.76% of hSyn-immunopositive cells expressed eNpHR3.0-EYFP. The promoter also provided high specificity near virus injection sites, as 98.9 ± 0.4% of eNpHR3.0-EYFP-expressing cells were hSyn-immunopositive (Fig 1C). To evaluate the physiological effects of eNpHR3.0 on VHP neuronal activity, optrode recordings in anesthetized rats were performed and it has been confirmed that continuous illumination of VHP neurons with 589-nm laser light inhibited neuronal firing in a temporally precise and reversible manner (Fig 1E). We compared the dEBC between PCP-eNpHR and PCP-EYFP group, as expected, we found that optogenetic inhibition of the bilateral VHP neurons caused significant improvements in the acquisition of long-dEBC, the blinking rates of PCP-eNpHR group were significantly higher than PCP-EYFP group (Figs 4E and 4I). In addition, data from long-latency time window show that optogenetic inhibition of the bilateral VHP neurons also improved the peak amplitude in session 7 (Fig 4J) and recovered the peak latency reduction to the equal level compared with control (Fig 4L). Together, these data provide evidence that the optogenetic inhibition of VHP neurons alleviated the defects of the long-dEBC induced by PCP administration.

## Discussion

SZ is an incurable mental disorder because its pathogenesis are still unclear. Recently, hippocampal DBS has been verified as being helpful in the treatment of experimental SZ[2, 7–10]. However, the target of cellular intervention during hippocampal DBS remains unknown. The neuronal cell bodies, the afferent neural fibers originating in remote brain areas, or even local glial cells are all possible candidates of DBS targets. Optogenetics has substantial advantages in both its higher accuracy and specificity in controlling the intended neural components. Here, we use an optogenetic approach to suppress the activities of VHP neurons in SZ-like rats induced by PCP to assess the involved hippocampal target. As CRs with short or middle latency in the long-dEBC paradigm have no protective effect against US punishment, which always occurred at the end of the CS, only eyeblink behavioural responses with long latency represent learned conditioned eyeblink behaviour here. By analyzing the CR incidence, which reflects the behavioral acquisition rate, the CR peak amplitude, which reflects the behavioral intensity, and the CR peak latency, which represents the behavioral timing accuracy, the present results show that (1) dEBC with a long CS duration of 3.3 seconds could be successfully established in rats; (2) PCP administration impaired both long-dEBC acquisition, which was reflected in a decrease in the CR incidence and CR peak amplitude, and long-dEBC timing, which was reflected by CR peak latency; and (3) inhibition of the bilateral VHP neurons alleviated both the impaired acquisition of long-dEBC by increasing CR%, CR peak amplitude, and the behavioral timing deficit by recovering CR peak latency. The present results suggest that the inhibition of local hippocampal neuronal cells may be a prominent effect of hippocampal DBS intervention in SZ. Our study also suggests that the pathogenesis of the SZ model established by systematical PCP administration is closely related to abnormally increased activity in the hippocampal network.

Excessive pathological ventral hippocampus-prefrontal projection excitation has been considered a primary candidate in the pathogenesis of PCP-induced SZ[14]. However, there are still contradicting reports. It has been reported that a subanaesthetic dose of ketamine (a similar NMDA receptor antagonista) inhibited hippocampus-mPFC excitatory synaptic transmission in unanaesthetized rats[27]. Although the present study is a preliminary work in which optogenetic inhibition was employed in all types of VHP neurons, not in specific subtypes, our experimental results support the hypothesis that increased activity in the hippocampal network may play a pivotal role in cognitive deficits of PCP-induced SZ. Moreover, it is thought that in SZ, excessive hippocampal output may also be responsible for excessive dopaminergic activity. Directly targeting hippocampal function could normalize abnormal dopamine neuron activity and restore deficits in cognitive function[7], thus, the success of optogenetics as a novel therapeutic approach for the treatment of SZ here may also be attributed to the mechanism of dopamine system regulation. However, our findings do not exclude other contributions to DBS in SZ. As local glial cells and afferent neural fibers originating from remote brain areas and projecting to the hippocampus have not been directly optogenetically modulated in this experiment, the possibility of direct or indirect effects of these components during DBS intervention in SZ cannot be ruled out. Further optogenetic work is needed to clarify the specific types of hippocampal neurons and the relevant circuits involved during this process.

Cognitive deficits are considered a key component of SZ dysfunctions[28]. EBC behavior is a thoroughly elucidated associative learning task and has been attached much importance recently in patients with SZ due to its minimal requirement for active motivation and attention[31–33]. Impaired simple dEBC performance indicating cerebellum dysfunction has been observed in several SZ studies[34, 35]. However, there are still conflicting results showing that patients with SZ appeared to show normal, even increased, levels of simple-dEBC acquisition and extinction, suggesting the intact cerebellar function in SZ[35]. Though the evidence for cerebellar dysfunction in SZ is less clear-cut, there is increasing evidence supporting hippocampal dysfunction in SZ[36–38]. Our previous study indicated that cynomolgus monkeys manifested prolonged CR deficits in tEBC, which is hippocampus-dependent, but not in simple dEBC, which is cerebellum-dependent, after the long-term administration of phencyclidine[39]. As long-dEBC is also hippocampus-dependent[24], the present results from a PCP rodent model demonstrate altered hippocampal function in SZ and show that optogenetically modulating hippocampal activity can alleviate cognitive dysfunctions in SZ.

In the current study, the time delay between CS onset and US onset was much longer than the typical interval of less than a second in the classical simple dEBC. To our knowledge, this is the first time that dEBC with such a long CS delay has been established. The present eyeblink data have been carefully analyzed to exclude the possibility of spontaneous blinking contamination, and there are no pre-existing deficits in general motor or sensory functions in PCP-administered rats because they exhibited similar CR%, CR amplitude and latency to those of controls during the early training sessions, indicating that with successive CS-US pairings, rats can actually learn the long-dEBC, perhaps as an “operant” reflex behaviour rather than as a classical one. Accurately timing the long-dEBC behaviour in the current study is much more difficult due to the long time delay, which is sufficient to allow the conscious involvement and voluntary control of eyeblink behaviour to protect from punishment pain. Further work is necessary to test whether the long-dEBC learning is an operant reflex behaviour. In addition, data from the long-latency time window showed that the optogenetic inhibition of bilateral VHP neurons could recover the peak latency reduction back to control levels. Given that the peak latency was significantly decreased after PCP administration, this result suggests the alleviating effect of optogenetics on behavioural timing deficits.

In contrast to electrical stimulation, which has the obvious limitation of recruiting multiple types of cells and/or axons, optogenetics technology has a great advantage over DBS in that it offers accurate and specific control of the intended neurons or their components. Several studies have used optogenetic technology to dissect relevant circuits or specific components of neural circuits implicated in SZ[40–42]. Thus, it has been proposed that with the current advanced optogenetic technology, it is time to seriously consider targeted interventions in the schizophrenic brain in both animal studies and clinical trials. The potential targets that are considered optional for intervention in the SZ brain should be part of the neural circuits that are involved in cognitive functions that are disturbed or, presumably damaged or malfuncitoning in SZ, such as the hippocampus, PFC and NAC[43]. Given that cognitive dysfunction is considered the primary cause of most behavioural disabilities associated with SZ[28–29], the restoration of EBC deficits by effective and selective local brain intervention may provide promising prospects for SZ alleviation.

## Supporting information

S1 Supporting Information (RAR)
